# Perinatal Resveratrol Therapy to Dioxin-Exposed Dams Prevents the Programming of Hypertension in Adult Rat Offspring

**DOI:** 10.3390/antiox10091393

**Published:** 2021-08-30

**Authors:** Chien-Ning Hsu, Chih-Hsing Hung, Chih-Yao Hou, Chi-I. Chang, You-Lin Tain

**Affiliations:** 1 Department of Pharmacy, Kaohsiung Chang Gung Memorial Hospital, Kaohsiung 83301, Taiwan; cnhsu@cgmh.org.tw; 2 School of Pharmacy, Kaohsiung Medical University, Kaohsiung 80756, Taiwan; 3 Department of Pediatrics, Kaohsiung Medical University Hospital, Kaohsiung 80756, Taiwan; pedhung@gmail.com; 4 Department of Seafood Science, National Kaohsiung University of Science and Technology, Kaohsiung 81157, Taiwan; chihyaohou@webmail.nkmu.edu.tw; 5 Department of Biological Science and Technology, National Pingtung University of Science and Technology, Pingtung 91201, Taiwan; 6 Department of Pediatrics, Kaohsiung Chang Gung Memorial Hospital, College of Medicine, Chang Gung University, Kaohsiung 83301, Taiwan; 7 Institute for Translational Research in Biomedicine, Kaohsiung Chang Gung Memorial Hospital, Kaohsiung 83301, Taiwan

**Keywords:** developmental origins of health and disease (DOHaD), gut microbiota, resveratrol, aryl hydrocarbon receptor, 3,3-dimethyl-1-butanol, 2,3,7,8-tetrachlorodibenzo-p-dioxin (TCDD), inflammation, T helper 17 cells, hypertension

## Abstract

Exposure to environmental chemicals during pregnancy and lactation is a contributing factor in gut microbiota dysbiosis and linked to programming of hypertension. 2,3,7,8-tetrachlorodibenzo-p-dioxin (TCDD), the most toxic dioxin, induces toxic effects by mediating aryl hydrocarbon receptor (AHR). Resveratrol, a potent antioxidant with prebiotic properties, can possess high affinity for AHR and protect against TCDD-activated AHR attack. We examined whether perinatal resveratrol therapy prevents offspring hypertension programmed by maternal TCDD exposure and whether its beneficial effects are related to reshaping gut microbiota and antagonizing AHR-mediated T helper 17 (TH17) cells responses using a maternal TCDD exposure rat model. Pregnant Sprague-Dawley rats were given a weekly oral dose of TCDD 200 ng/kg for four doses (T), 50 mg/L of resveratrol in drinking water (CR), TCDD + resveratrol (TR), or vehicle (C) in pregnancy and lactation periods. Male offspring (*n* = 7–8/group) were sacrificed at the age of 12 weeks. Perinatal TCDD exposure caused elevated blood pressure in adult male offspring, which resveratrol supplementation prevented. Additionally, the TCDD-induced programming of hypertension is coincided with the activation of AHR signaling, TH17-induced renal inflammation, and alterations of gut microbiota compositions. Conversely, TCDD-mediated induction of AHR signaling and TH17 responses were restored by maternal resveratrol supplementation. Furthermore, maternal resveratrol supplementation prevented the programming of hypertension and was related to increased genera *Bacteroides*, *ASF356*, and *Lachnoclostridium*. Taken together, these results suggest that the interplay between gut microbiota, AHR-mediated TH17 responses, and renal inflammation in the gut and kidneys may play an important role in the action of resveratrol against TCDD-induced programming of hypertension.

## 1. Introduction

Hypertension is a complex disorder with various factors contributing to its prevalence. It remains one of the most significant causes of premature death worldwide [1]. Hypertension can take its origin in early life. It is assumed that many maternal exposures at early stages of fetal development contribute to the risk of later development of chronic disease [2], and is now known as the Developmental Origins of Health and Disease (DOHaD) [3]. Over the years, it has become clear that adult disease of developmental origins could be preventable by shifting interventions from adulthood to earlier stage referred to as reprogramming [4,5].

The kidney is the major regulatory organ for maintaining blood pressure (BP). Development of the kidney can be adapted in utero in response to suboptimal conditions, resulting in renal programming [6]. More and more evidence is emerging highlighting the important role of renal programming in the development of hypertension [4,5,6,7]. A wide variety of maternal insults can affect developing kidney, resulting in renal programming and consequently adulthood hypertension [8]. These include maternal undernutrition, overweight, maternal illness, drug use, infection, inflammation, exposure to environmental chemicals, etc. [8]. Many studies have reported the adverse effects that occur in later life following exposure to environmental chemicals during kidney development [9].

Dioxins are persistent environmental pollutants [10]. Dioxins are emitted commonly from anthropogenic sources like manufacturing of pesticides, bleaching of wood pulp and waste incineration. Dioxins continue to raise concern because of their highly toxic potential [10]. Among them, 2,3,7,8-tetrachlorodibenzo-p-dioxin (TCDD) is the most extensively studied and toxic dioxin. The oral lethal dosage 50% (LD50) values of TCDD fall in a range of 300 to 1000 mg/kg for rats, mice, guinea pigs, and rabbits; its immediately dangerous to life or health (IDLH) concentration was 250 mg/m^3^ [11]. Prior research has shown that exposure to TCDD during pregnancy was associated with negative health outcomes for offspring [12,13]. The majority of biological functions of TCDD leading to toxic effects are mediated by aryl hydrocarbon receptor (AHR) [14]. Binding of TCDD results in nuclear translocation of the AHR, dissociation from the chaperone proteins, heterodimerization with Aryl hydrocarbon Receptor Nuclear Translocator (ARNT). The AHR/ARNT heterodimer binds to the promoter region of various AHR target genes to initiate their transcription [14]. Our previous study demonstrated that perinatal TCDD exposure exacerbated high-fructose diet-induced hypertension in male adult offspring, which was associated with the activation of AHR signaling pathway [15].

Conversely, competitive binding of AHR to dietary polyphenols possessing high affinity for AHR may protect the organism from TCDD-activated AHR attack and decreases the risk of various diseases [16]. Resveratrol is a natural polyphenol, can have antioxidant and prebiotics properties, and provides a wide spectrum of beneficial effects on human health [16,17,18]. More importantly, resveratrol has been used as a reprogramming intervention with therapeutic potential in the developmental origins of adult disease [19]. Resveratrol was most commonly given in drinking water at the dose of 50 mg/L [19,20], followed by a chow diet supplemented with resveratrol (4 g/kg diet) [19,21]. As reviewed elsewhere [19], maternal resveratrol treatment has shown benefits against hyperglycemia, insulin resistance, obesity, hyperlipidemia, and hypertension programmed by various maternal insults in adult offspring.

The current study was undertaken to interrogate whether perinatal TCDD exposure produces the programming of hypertension in adult life by mediating the AHR signaling pathway in adult offspring and whether maternal resveratrol supplementation was protected. Additionally, impaired nitric oxide (NO) pathway and gut microbiota dysbiosis are linked to programming of hypertension [22,23]. Microbiota-generated AHR agonists can stimulate T helper 17 (TH17) cells to secrete interleukin-17A (IL-17A) and promote inflammation [24]. Considering resveratrol can enhance NO bioavailability [19] and act like probiotics to modulate gut microbiota and reduce TH17 response [25], we also delineated that the molecular mechanisms underpinning the beneficial effects of resveratrol were related to restoration of AHR signaling, TH17-mediated inflammation, NO pathway, and gut microbiota.

## 2. Materials and Methods

### 2.1. Animals

The parent stocks of male and female Sprague-Dawley (SD) rats were purchased from BioLASCO (Taipei, Taiwan). Rats were allowed to acclimatize in a temperature-controlled room at 22 ± 1 °C with controlled humidity at 55 ± 5% and light (12:12 light-dark cycle, light on from 08:00) in an AAALAC-International accredited animal facility at our hospital. In view of the fact that BP tended to be significantly higher for males in younger age groups than females [26], after birth, only male offspring were used in subsequent experiments. We culled each litter to eight pups to standardize maternal care and milk quality.

Male offspring were allocated into four groups (*n* = 8 per group): control rats (C), rats treated with resveratrol (R), rats exposed to TCDD (T), and rats received administration of TCDD and resveratrol (TR). To construct a TCDD exposure model, pregnant dams received an oral dose of TCDD (Sigma-Aldrich, St. Louis, MO, USA) at 200 ng/kg body weight (BW) or vehicle (corn oil, 4 mL/kg BW) on gestational days 14 and 21 and days 7 and 14 after birth to cover the period of kidney development. The weekly dose of TCDD used here was in accordance with prior research showing the half-life of TCDD in rats is approximately 3 weeks [27]. Half of control and TCDD exposure pregnant rats received 50 mg/L of resveratrol in drinking water during pregnancy and lactation periods. The dose was selected based on our prior work [28].

We used the CODA noninvasive BP system (Kent Scientific Corporation, Torrington, CT, USA) for measurement of BP according to our previous protocol [28]. The CODA rat tail-cuff system was designed to allow accurate BP measurement using volume pressure recording sensor technology in rats. Fecal samples were collected, frozen, and placed into a −80 ◦C freezer. Rat were killed at 12 weeks with an i.p. overdose of pentobarbital. Blood samples, kidneys, and fecal samples were collected.

All animal experiments were conducted in accordance with the Guide for the Care and Use of Laboratory Animals of the National Institutes of Health and approved by the Institutional Animal Care and Use Committee of Chang Gung Memorial Hospital, No. 2020061202.

### 2.2. Quantitative Real-Time Polymerase Chain Reaction (qPCR)

We determined renal mRNA expression of AHR and related gene by qPCR, following previously described methods [15]. These included AHR, aryl hydrocarbon receptor nuclear translocator (ARNT), aryl hydrocarbon receptor repressor (AHRR), and cytochrome P450 CYP1A1 (CYP1A1). RNA was extracted from each offspring’s kidney cortex. We used iCycler iQ Real-Time PCR Detection System (Bio-Rad, Hercules, CA, USA) and Quantitect SYBR Green PCR Reagents kit (Qiagen, Valencia, CA, USA) to perform two-step quantitative real-time PCR. The R18S reference gene was used as the internal control as its constant expression across all samples. All samples were assayed in duplicate. We calculated relative gene expression using the comparative threshold cycle (Ct) method. The fold change was established by calculating 2^−ΔΔCt^ for experimental versus reference samples. The sequence of the primers used is provided in Table 1.

### 2.3. Gut Microbiota Compositions

Stool samples were analyzed with metagenomics using the methods published previously [15]. According to the manufacturer’s protocol (the Biotools Co., Ltd., Taipei, Taiwan), the variable region 4 (V4) of small subunit rRNA (16S rRNA) gene was PCR-amplified and amplicons were mixed together for sequencing using Illumina Miseq platform (Illumina, San Diego, CA, USA). Illumina sequence data were carried out using QIIME version 1.9.1. Sequences (Illumina, San Diego, CA, USA). The high-quality sequences were clustered into operational taxonomic units (OTUs) at a 97% sequence similarity using the USEARCH algorithm. Based on a representative sequence alignment with Fast-Tree, the phylogenetic relationships were constructed. Statistical analysis of community structure at each classification level were performed. We investigated the α-/β-diversity patterns of microbial communities [29]. Alpha diversity was measured by the phylogenetic diversity (PD) whole tree (richness) and Shannon (evenness) diversity indexes. To assess the β-diversity of gut microbiota, the Analysis of Similarities (ANOSIM) and the partial least squares discriminant analysis (PLS-DA) were performed. The linear discriminant analysis effect size (LEfSe) was used to screen biomarkers that can most likely explain the differences within each group. The threshold on logarithmic score (LDA) for discriminative features was set to three.

### 2.4. Measurement of Cytokines in the Kidneys

The cytokines interleukin IL-2, IL-6, IL-10, IL-17A, IL-17F, IL-22, and interferon-γ (IFN-γ) were measured in the kidney cortex homogenates using a LEGENDplex Custom 9plex cytokine panel kit (BioLegend, San Diego, CA, USA) according to the manufacturer’s instructions. The samples were analyzed on a BD FACS Canto II flow cytometer (BD Biosciences, San Jose, CA, USA). Briefly, the tissue samples were diluted in the assay buffer and measured in duplicate. The color code of each bead identifies the target protein being assayed, while the fluorescent intensities on the beads measure target concentration. The data was quantified by LEGENDplex analysis software (BioLegend, San Diego, CA, USA). To adjust measurements for differences in total protein content of the samples, values are expressed in picograms per total gram of protein.

### 2.5. Analysis of NO Pathway

We used high-performance liquid chromatography (HP series 1100; Agilent Technologies Inc., Santa Clara, CA, USA) with fluorescence detection of O-phthalaldehyde/3-mercaptopropionic acid (OPA/3MPA) derivatives to analyze parameters related to NO pathway [15]. These NO-related parameters included l-citrulline (the precursor of l-arginine), l-arginine (substrate for NO synthesis), asymmetric and symmetric dimethylarginine (ADMA & SDMA; inhibitors of NO synthase). Homoarginine (Sigma-Aldrich, St. Louis, MO, USA) was used as the internal standard.

### 2.6. Statistical Analysis

Mean ± SEM was routinely used. Comparisons within four groups were analysis by one-way analysis of variance (ANOVA) followed by a Tukey’s post hoc test. The statistical significance of differences in bacterial composition among the different samples was assessed by either the Wilcox or the Kruskal–Wallis test. *p* values were deemed significant if less than 0.05. We used the Statistical Package for the Social Sciences software (SPSS Inc., Chicago, IL, USA) to analyze all data.

## 3. Results

### 3.1. Morphological Values and Blood Pressures

One pup of the C group was dead in the first week of life. The T and TR group had a lower body weight (BW) than that in the C group (Table 2). The kidney weight was comparable among the four group, while TCDD caused an increase of kidney weight-to-BW ratio in the T group. We observed that systolic and diastolic BPs (SBP and DBP) and mean arterial pressure were significantly increased in the T group at 12 weeks of age. The TCDD-induced increases in SBP were prevented by the resveratrol therapy.

### 3.2. AHR Signaling Pathway

**Figure 1:**
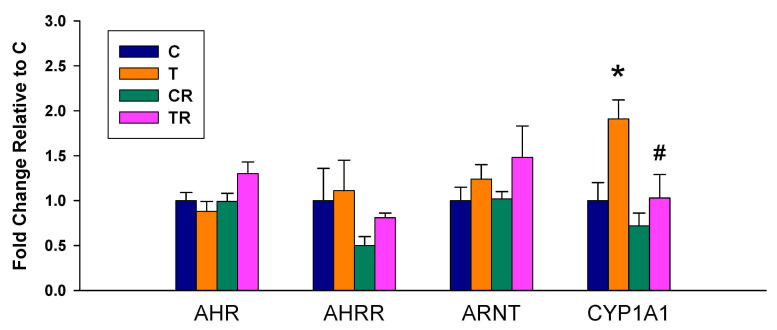
Effect of 2,3,7,8-tetrachlorodibenzo-p-dioxin (T) and resveratrol (R) on the mRNA expression of acryl hydrocarbon receptor (AHR) signaling pathway in offspring kidneys. C = control rats received vehicle; T = rats received weekly oral dose of TCDD 200 ng/kg for four doses; CR = control rats received 50 mg/L of resveratrol in drinking water during pregnancy and lactation; TR = rats received TCDD and resveratrol treatment; AHR= aryl hydrocarbon receptor; AHRR = aryl hydrocarbon receptor repressor; ARNT = aryl hydrocarbon receptor nuclear translocator; CYP1A1 = cytochrome P450 CYP1A1. * *p* < 0.05 vs. C; ^#^ *p* < 0.05 vs. T.

We observed there was a significant increase in mRNA expression of CYP1A1 in the T group vs. the C group, which was prevented by the resveratrol therapy (Figure 1). Whereas no differences in renal mRNA of AHR, AHRR, and ARNT were detected between any of the groups.

### 3.3. Cytokine Concentrations in the Kidneys

In the kidney tissues (Table 3), the levels of IL-6, IL-17A, and IFN-γ were elevated in the T group. However, these TCDD-induced increases were restored by resveratrol therapy. Renal IL-2 and IL-22 level was higher in the T group than that in the C group. No differences in IL-10 and IL-17F were detected between any of the groups.

### 3.4. Gut Microbiota Composition

**Figure 2:**
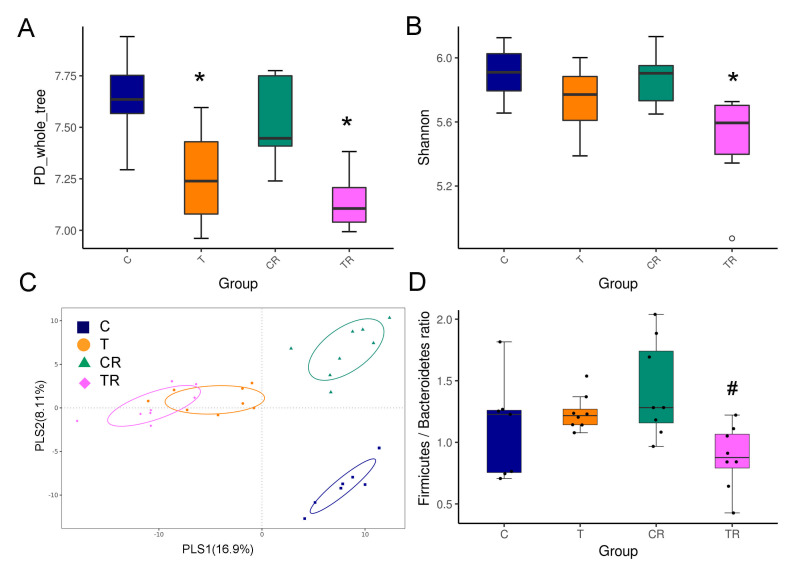
Effect of 2,3,7,8-tetrachlorodibenzo-p-dioxin (T) and resveratrol (R) on the gut microbiome in 12-week-old offspring. α-diversity measured by (**A**) PD whole tree and (**B**) Shannon index. (**C**) β-diversity measured by partial least squares discriminant analysis (PLS-DA). (**D**) The *Firmicutes* to *Bacteroidetes* (F/B) ratio. C = control rats received vehicle; T = rats received weekly oral dose of TCDD 200 ng/kg for four doses; CR = control rats received 50 mg/L of resveratrol in drinking water during pregnancy and lactation; TR = rats received TCDD and resveratrol treatment. * *p* < 0.05 vs. C; ^#^ *p* < 0.05 vs. T.

To further elucidate the mechanism of the beneficial effect of resveratrol therapy, we investigated the impact of resveratrol on the gut microbiota composition. As shown in Figure 2A, the PD whole tree index was significantly lower in the T and TR group compared to controls, suggesting resveratrol treatment did not restore bacterial richness. The Shannon diversity index was used to determine α-diversity and it was found that TR group was lower than that in the C group (Figure 2B). The score plots of PLS-DA analysis showed a clear separation between each group (Figure 2C). Likewise, the ANOSIM analysis within each group did reach significance (All *p* < 0.01). Our data suggests that the gut microbiota composition was significantly altered by TCDD and resveratrol, whereas resveratrol might have only a negligible impact on richness and evenness in the TR group.

At the phylum level, resveratrol treatment caused a lower relative abundance of *Firmicutes* and higher abundance of *Bacteroidetes* in the TR group vs. T group (Both *p* < 0.05), which was further quantified by calculating the *Firmicutes* to *Bacteroidetes* (F/B) ratio (Figure 2D). Hypertension has been associated with a high F/B ratio [23]. We observed that resveratrol therapy reduced the F/B ratio in the TR group compared to the T group.

**Figure 3:**
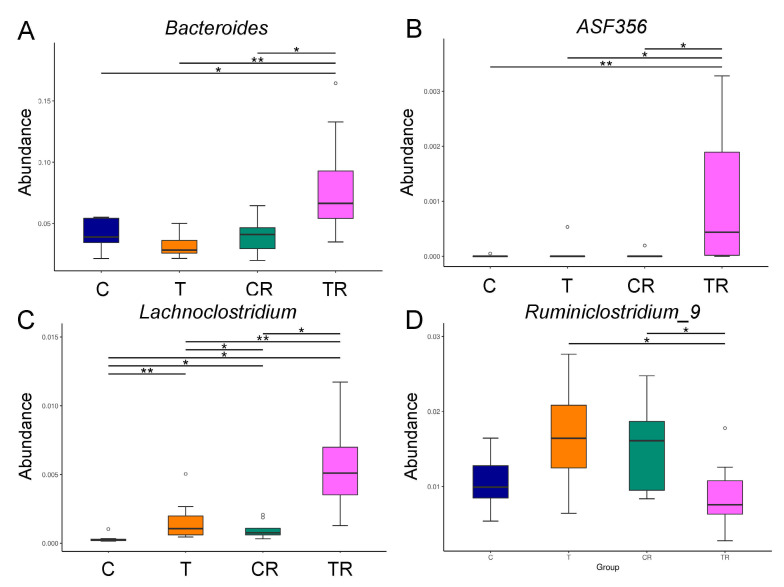
Effect of 2,3,7,8-tetrachlorodibenzo-p-dioxin (T) and resveratrol (R) on the gut microbiome in 12-week-old offspring. Relative abundance of the genera (**A**) *Bacteroides*, (**B**) *ASF356*, (**C**) *Lachnoclostridium*, and (**D**) *Ruminiclostridium_9*. C = control rats received vehicle; T = rats received weekly oral dose of TCDD 200 ng/kg for four doses; CR = control rats received 50 mg/L of resveratrol in drinking water during pregnancy and lactation; TR = rats received TCDD and resveratrol treatment. * *p* < 0.05; ** *p* < 0.01.

At the genus level, the abundance of *Bacteroides* and *ASF356* was highest in the TR group (Figure 3A,B). Figure 3C shows *Lachnoclostridium* of the genus-level was significantly greater in the T and CR group vs C group, with the greatest level in the TR group. Additionally, the abundance of genus *Ruminiclostridium_9* was lower in the TR group than that in the T group (Figure 3D).

**Figure 4:**
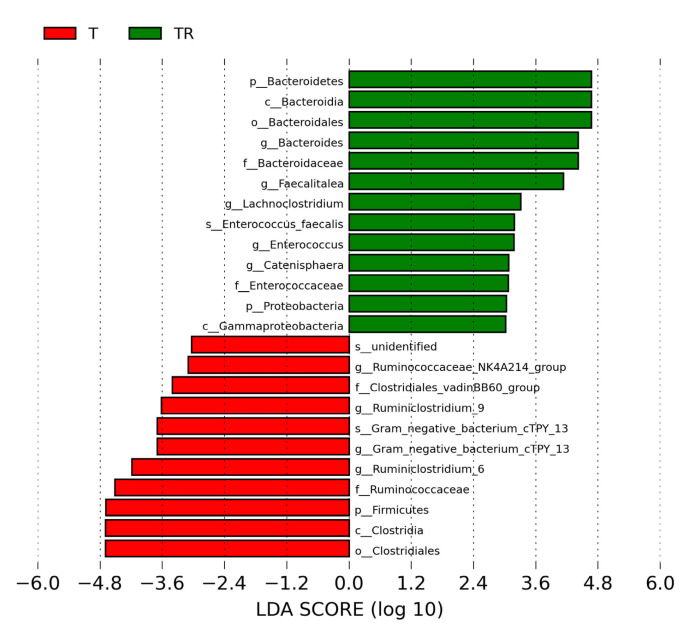
Effect of 2,3,7,8-tetrachlorodibenzo-p-dioxin (T) and resveratrol (R) on the gut microbiome in 12-week-old offspring. Most enriched and depleted bacterial taxa in the T (red) versus TR group (green) measured by linear discriminant analysis effect size (LEfSe) are shown. The threshold on the linear discriminant is set to 3. T = rats received weekly oral dose of TCDD 200 ng/kg for four doses; TR = rats received TCDD and resveratrol treatment.

We next used LEfSe analysis to identify bacteria where the relative abundance was significantly different between the T and TR group. Figure 4 illustrates there was a greater abundance of genera *Bacteroides, Faecalitalea, Enterococcus,* and *Lachnoclostridium*; whereas a lower abundance of *Ruminiclostridium_6* and *Ruminiclostridium_9* in the TR group vs. the T group. These could be used as microbial markers for resveratrol-induced gut microbiome remodeling.

### 3.5. Plasma NO-Related Parameters

We next analyzed NO-related parameters as NO has also been implicated in programming of hypertension [22]. Table 4 illustrates resveratrol therapy caused a higher concentration of plasma l-Arginine but a lower SDMA the CR group compared to the C group. We did not detect any differences of l-Citrulline and ADMA levels in the plasma among the four groups. However, the l-Arginine-to-ADMA ratio was higher in the CR group compared to controls (Table 4). These findings suggest resveratrol increased NO bioavailability in the control offspring, but not in TCDD-exposed offspring.

## 4. Discussion

This study extends our knowledge of beneficial effects of resveratrol on hypertension programmed by perinatal TCDD exposure with particular emphasis on AHR signaling and gut microbiota. The most significant findings of the current study are: (1) maternal supplementation with resveratrol throughout pregnancy and lactation prevents the programming of hypertension in adult offspring perinatally exposed to TCDD; (2) the programming of hypertension in TCDD-exposed offspring is attributed to the activation of AHR signaling, TH17-induced renal inflammation, and alterations of gut microbiota compositions; (3) the TCDD-mediated induction of AHR/CYP1A1 and TH17-mediated renal inflammation could be significantly restored by maternal supplementation of resveratrol; (4) TCDD and resveratrol shaped the gut microbiome differentially, resulting in distinctive boundaries of gut enterotypes; and (5) resveratrol therapy prevented programmed hypertension was coincided with increased genera *Bacteroides*, *ASF356*, and *Lachnoclostridium*.

The protective effect of resveratrol supplementation could be due to it antagonizing AHR-mediated TH17-driven renal inflammation. Prior research suggests the AHR signaling pathway is involved in TCDD-induced hypertension [30]. Our previous study showed resveratrol protected the programming of hypertension relevant to antagonizing the AHR signaling pathway in a maternal high-fat diet combined bisphenol A exposure model [31]. Considering activation of the AHR/CYP1A1 axis induces vasoconstriction [26], resveratrol suppress renal CYP1A1 expression might, at least in part, contribute to its beneficial effects against TCDD-induced hypertension. However, when comparing our results to those of prior research using a combined prenatal dexamethasone (DEX) and TCDD exposure [32], it must be pointed out that resveratrol mediated not only CYP1A1 but also AHRR in the DEX + TCDD model. Although similar pattern of BP-lowering effect by resveratrol was obtained in the TCDD as well as in the DEX + TCDD model, these findings suggest the protective mechanism of resveratrol may differ between the one-hit and two-hit model.

Among several mechanisms proposed behind programming of hypertension, renal inflammation represents a key pathophysiological process [4,5,6,7,8]. Pro-inflammatory cytokines contribute to a state of persistent low-grad inflammation. Of the pro-inflammatory cytokines, increased IL-17A level due to activating TH17 axis has linked AHR signaling to dysbiotic gut microbiota and inflammation [20]. Prior research suggests that dysbiotic gut microbiome could promote TH17 cell activation and stimulate the release of IL-6, IL-17A, and IFN-γ, leading to hypertension [33]. In line with this postulation, our results demonstrated that the increases in BP of adult offspring perinatally exposed to TCDD was accompanied with a parallel increase in renal IL-6, IL-17A, and IFN-γ levels, indicating an activation of the TH17 axis and renal inflammation.

The beneficial effects of resveratrol are coincided with reshaping gut microbiome by reducing F/B ratio and augmenting certain microbes that can inhibit TH17 responses. In support of previous research indicating that high F/B ratio is associated with hypertension [23], we found resveratrol therapy reduced this ratio and BP concurrently. Gut microbiota can shape the immune system by regulating T helper cell lineage differentiation [34]. Certain bacteria, like *Bacteroides* and altered Schaedler flora (ASF), induce regulatory T (Treg) cell differentiation and reciprocally diminish TH17 responses [34]. Here, we show that resveratrol treatment augments the genera abundance of *Bacteroides* and *ASF356* (a member of the ASF) and inhibits TH17 responses concurrently in the TR group. Our results provide important information on how resveratrol alters gut microbes and restores the balance of TH17/Treg to prevent the programming of hypertension. Additionally, low level of genus *Lachnoclostridium* but high abundance of *Ruminiclostridium* has been identified in hypertensive patients [35,36]. According to our data, resveratrol treatment increased *Lachnoclostridium* and decreased *Ruminiclostridium_9*. Thus, whether the beneficial effects of resveratrol attributed to alterations of these microbes deserves further clarification. Moreover, increased NO bioavailability is a protective mechanism reported as underlying resveratrol against the programming of hypertension [19,28]. Our current study failed to identify the differences of NO-related parameters between the T and TR group, despite resveratrol increasing NO bioavailability in the controls.

A potential limitation of the present study is the inability to longitudinally analyze gut microbiome dynamics at different developmental stages. It is possible that alterations of gut microbiome observed at 12-week-old TCDD-exposed offspring happened as a result of hypertension. More attention needs to be paid to analyze how gut microbiome interacts with the host at the early developmental stage to help elucidate the causal relationship and explore the underpinning programmed processes. Next, in view of diverse biological activities of resveratrol, its beneficial effects could be attributed to other mechanisms without a holistic approach. Last, we did not examine other dosages of resveratrol administration, thus, whether its reprogramming effects on TCDD-induced hypertension exist in a dose-dependent manner remains to be determined.

## 5. Conclusions

In summary, the present study provides novel evidence to suggest that resveratrol therapy is a potential reprogramming strategy targeting the gut–kidney axis to protect against the programming of hypertension. Although our results provide novel mechanistic aspects of resveratrol, these preclinical results await clinical translation.

## Figures and Tables

**Table 1:** Primer sequences used for qPCR analysis.

Gene	Accession Number	Forward	Reverse
AHR	AM902286.1	GTCCTCAGCAGGAACGAAAG	CCAGGGAAGTCCAACTGTGT
AHRR	AY367561.1	CAGCAACATGGCTTCTTTCA	TGAAGCACTGCATTCCAGAC
ARNT	NM_012780.3	GTCTCCCTCCCAGATGATGA	GCTGGTAGCCAACAGTAGCC
CYP1A1	NM_012540.3	GCACTCTGGACAAACACCTG	ATATCCACCTTCTCGCCTGG
R18S	M11188.1	GCCGCGGTAATTCCAGCTCCA	CCCGCCCGCTCCCAAGATC

AHR = aryl hydrocarbon receptor, AHRR = aryl hydrocarbon receptor repressor, ARNT = aryl hydrocarbon receptor nuclear translocator, CYP1A1 = cytochrome P450 CYP1A1, R18S = 18S ribosomal RNA.

**Table 2:** Morphological values and blood pressures.

Groups	C	T	CR	TR
	*N* = 7	*N* = 8	*N* = 8	*N* = 8
Body weight (BW) (g)	415 ± 20	332 ± 7 *	416 ± 25	355 ± 15 *
Left kidney weight (g)	1.86 ± 0.1	1.73 ± 0.05	1.69 ± 0.1	1.78 ± 0.07
Left kidney weight/100 g BW	0.45 ± 0.02	0.52 ± 0.01 *	0.41 ± 0.01	0.50 ± 0.03
Systolic blood pressure (mmHg)	129 ±1	142 ± 2 *	130 ± 0	133 ± 0 ^#^
Diastolic blood pressure (mmHg)	80 ± 2	87 ± 3 *	86 ± 2	85 ± 1
Mean arterial pressure (mmHg)	97 ± 1	105 ± 2 *	100 ± 1	101 ± 1

C = control rats received vehicle; T = rats received weekly oral dose of TCDD 200 ng/kg for four doses; CR = control rats received 50 mg/L of resveratrol in drinking water during pregnancy and lactation; TR = rats received TCDD and resveratrol treatment. * *p* < 0.05 vs. C; ^#^ *p* < 0.05 vs. T.

**Table 3:** Kidney tissue cytokine levels.

Groups	C	T	CR	TR
	*N* = 7	*N* = 8	*N* = 8	*N* = 8
IL-2 (pg/g protein)	4283 ± 653	9075 ± 1270 *	6391 ± 1037	5620 ± 317
IL-6 (pg/g protein)	6864 ± 1198	14,948 ± 1805 *	10,724 ± 1858	5373 ± 557 ^#^
IL-10 (pg/g protein)	2040 ± 1072	10,390 ± 5561	562 ± 279	3136 ± 961
IL-17A (pg/g protein)	5529 ± 870	11,332 ± 1411 *	8151 ± 1152	7293 ± 303 ^#^
IL-17F (pg/g protein)	2293 ± 336	3262 ± 388	2394 ± 179	3095 ± 187
IL-22 (pg/g protein)	1979 ± 273	3208 ± 404 *	2332 ± 218	2838 ± 141
IFN-γ (pg/g protein)	3725 ± 586	8545 ± 1128 *	5668 ± 982	4829 ± 337 ^#^

C = control rats received vehicle; T = rats received weekly oral dose of TCDD 200 ng/kg for four doses; CR = control rats received 50 mg/L of resveratrol in drinking water during pregnancy and lactation; TR = rats received TCDD and resveratrol treatment. IL = interleukin; IFN-γ = interferon-γ. * *p* < 0.05 vs. C; ^#^ *p* < 0.05 vs. T.

**Table 4:** Plasma NO-related parameters.

Groups	C	T	CR	TR
	*N* = 7	*N* = 8	*N* = 8	*N* = 8
l-Citrulline (μM)	71.8 ± 3.5	65 ± 3.8	73.9 ± 2.3	63.5 ± 5.3
l-arginine (μM)	134.9 ± 5.7	149.6 ± 7.9	189.3 ± 9.3 *	159.9 ± 5.7
ADMA (μM)	1.56 ± 0.06	1.6 ± 0.08	1.37 ± 0.07	1.59 ± 0.08
SDMA (μM)	1.35 ± 0.03	1.69 ± 0.08	1.28 ± 0.09 *	1.32 ± 0.03
l-arginine-to-ADMA ratio (μM/μM)	87.5 ± 6	96.3 ± 8.4	141.2 ± 11.1 *	102.4 ± 6.8

C = control rats received vehicle; T = rats received weekly oral dose of TCDD 200 ng/kg for four doses; CR = control rats received 50 mg/L of resveratrol in drinking water during pregnancy and lactation; TR = rats received TCDD and resveratrol treatment. * *p* < 0.05 vs. C.

## Data Availability

Data is contained within the article.
